# Comparison of the outcome between cervical adenocarcinoma and squamous cell carcinoma patients with adjuvant radiotherapy following radical surgery: SGSG/TGCU Intergroup Surveillance

**DOI:** 10.3892/mco.2013.112

**Published:** 2013-05-09

**Authors:** MUNEAKI SHIMADA, RYUICHIRO NISHIMURA, TAKAMITSU NOGAWA, MASAYUKI HATAE, KAZUHIRO TAKEHARA, HIDEKAZU YAMADA, HIROHISA KURACHI, YOSHIHITO YOKOYAMA, TORU SUGIYAMA, JUNZO KIGAWA

**Affiliations:** 1 Department of Obstetrics and Gynecology, Tottori University School of Medicine, Yonago, Tottori 683-8504;; 2 Department of Gynecologic Oncology, Hyogo Cancer Center, Akashi, Hyogo 673-8558;; 3 Department of Gynecology, National Hospital Organization Shikoku Cancer Center, Matsuyama, Ehime 791-0280;; 4 Department of Obstetrics and Gynecology, Kagoshima City Hospital, Kagoshima, Kagoshima 892-8580;; 5 Department of Obstetrics and Gynecology, National Hospital Organization Kure Medical Center, Kure, Hiroshima 737-0023;; 6 Department of Obstetrics and Gynecology, Fukushima Medical University School of Medicine, Fukushima, Fukushima 960-1295;; 7 Department of Obstetrics and Gynecology, Yamagata University, Yamagata, Yamagata 990-9585;; 8 Department of Obstetrics and Gynecology, Hirosaki University Graduate School of Medicine, Hirosaki, Aomori 036-8562;; 9 Department of Obstetrics and Gynecology, Iwate Medical University, School of Medicine, Morioka, Iwate 020-8505;; 10 Cancer Center, Tottori University Hospital, Yonago, Tottori 683-8504, Japan

**Keywords:** adenocarcinoma, squamous cell carcinoma, uterine cervix, radiotherapy

## Abstract

The efficacy of radiotherapy (RT) for adenocarcinoma (AC) is controversial, although patients with AC of the uterine cervix are treated in a similar manner to those with squamous cell carcinoma (SCC). This retrospective study was conducted to evaluate the efficacy of adjuvant RT for patients with AC compared to those with SCC following radical hysterectomy. A total of 820 patients with stage IB-IIB cervical cancer, who underwent type III radical hysterectomy between 1997 and 2003, were retrospectively examined; the sample included 280 patients with AC and 540 with SCC. A total of 139 patients with AC and 327 with SCC underwent adjuvant treatment. The histological type did not affect the outcome for patients with stage I disease; however, stage II patients with AC exhibited a significantly worse 5-year overall survival (OS) rate compared to those with SCC. Patients with SCC exhibited significantly higher lymph node involvement compared to those with AC in stage IB1; however, there were no differences between stages IB2 and II. Among patients with lymph node involvement, patients with AC exhibited a significantly worse 5-year survival rate compared to those with SCC (46.4 vs. 72.3%, respectively; P=0.0005). Among patients receiving adjuvant RT, those with AC recurred more frequently compared to those with SCC, particularly in the pelvic cavity, including the vaginal stump and/or pelvis (24.6 vs. 10.5%, respectively; P= 0.0022). By contrast, the histological type did not affect the incidence of recurrence in paraaortic lymph nodes and/or distant recurrence. In conclusion, RT may not suffice as an adjuvant treatment for patients with cervical AC following radical hysterectomy.

## Introduction

The standard treatment for patients with International Federation of Gynecology and Obstetrics (FIGO) stage IB-II cervical cancer is radical hysterectomy and/or radiotherapy (RT), including concurrent chemoradiotherapy (CCRT). In Japan, the majority of gynecologic oncologists select radical hysterectomy for patients with stage IB-II cervical cancer (1). By contrast, the National Comprehensive Cancer Network (NCCN) clinical practice guidelines recommend radical hysterectomy for patients with IA2, IB1 and IIA1 and CCRT for patients with stage IB2, IIA2 and IIB cervical cancer (2).

A previous study by the Gynecologic Oncology Group (GOG) demonstrated that adjuvant pelvic RT following radical hysterectomy reduced the number of recurrences in stage IB patients with intermediate risk factors (3). In addition, another GOG study (GOG109/SWOG 8797/RTOG91-12) suggested that RT with concurrent cisplatin-containing chemotherapy (CT) was more effective for stage IA2-IIA patients with pelvic lymph node involvement, parametrial extension or compromised surgical margin compared to RT alone following radical hysterectomy (4). Therefore, the NCCN clinical practice guidelines recommend adjuvant RT including CCRT for cervical cancer patients with pathological risk factors following radical hysterectomy (2).

Since the incidence of adenocarcinoma (AC) of the uterine cervix has increased from ∼12 to 24% of cervical cancer cases over the past 24 years (5), effective therapeutic strategies for AC are required. Whether the prognosis of patients with cervical cancer is dependent on histological type remains controversial (6–11). A previous GOG study of 813 patients with stage IB cervical cancer, 645 of whom had squamous cell carcinoma (SCC) and 168 AC, including adenosquamous cell carcinoma (ASCC), demonstrated that there were no statistically significant differences regarding the recurrence-free interval among histological types (6). The NCCN clinical practice guidelines also suggest that AC may be effectively treated in a similar manner to SCC (2). In a previous study, we reported that stage II patients with AC had a significantly worse prognosis compared to those with SCC, although the survival of stage IB patients did not differ between AC and SCC (12). Additionally, findings of previous studies suggested that the radiosensitivity of AC may be lower compared to that of SCC (13–15). Consequently, adjuvant RT, which is recommended as the standard adjuvant treatment for high- or intermediate-risk patients with cervical cancer, may be of limited value for patients with AC following radical hysterectomy. This retrospective study was conducted to evaluate the efficacy of adjuvant RT for patients with AC compared to those with SCC following radical hysterectomy.

## Patients and methods

### Patient data

A total of 820 patients with FIGO stage IB-IIB cervical cancer, who underwent type III radical hysterectomy at 10 institutes (Hyogo Cancer Center, Kagoshima City Hospital, National Hospital Organization Shikoku Cancer Center, National Hospital Organization Kure Medical Center, Fukushima Medical University, Yamagata University, Tohoku University School of Medicine, Hirosaki University School of Medicine, Iwate Medical University and Tottori University Hospital) between 1997 and 2003, were enrolled in this study. Data were collected from patient medical records. The retrospective study protocol was approved by the Institutional Review Board of each institution. There were 540 SCC and 280 AC patients. The indications for adjuvant treatment were as follows: pelvic lymph node involvement, parametrial extension, deep stromal invasion, vessel permeation and a compromised surgical margin; however, the indications for adjuvant treatment were not identical among the 10 institutes. The chemotherapeutic regimens and number of cycles were also decided on by each institution, although the majority of adjuvant CT was platinum-based combination CT.

### Statistical analysis

Patient survival distribution was calculated using the Kaplan-Meier method. The significance of the survival distribution in each group was assessed by the log-rank test. The Chi-square test was used to compare any associations between prognostic factors. P<0.05 was considered to indicate a statistically significant difference.

## Results

### Patient data

A total of 354 patients, 141 of whom had AC and 213 SCC, underwent radical hysterectomy alone (Table I). The remaining 466 patients, of whom 139 had AC and 327 SCC, received adjuvant treatment following radical hysterectomy. The distribution of patients with SCC receiving adjuvant treatment was significantly higher compared to those with AC (60.6 vs. 49.6%, respectively; P= 0.0028). Out of the 139 AC patients, 69 received RT alone or CCRT with weekly cisplatin (CDDP), 54 received CT alone and 16 received concomitant RT and CT. Out of the 327 SCC patients receiving adjuvant treatment, 258 received RT alone or CCRT with weekly CDDP, 36 received CT alone and 33 received concomitant RT and CT.

The 5-year overall survival (OS) rate for patients with AC and SCC was 87.4 and 83.4%, respectively (P=0.2509). The 5-year OS for stage IB1 was 92.0% in AC, 94.7% in SCC and for stage IB2 survival was 75.5% in AC and 74.2% in SCC. Patients with AC exhibited a significantly worse outcome compared to those with SCC in stage II (stage IIA: 54.5 vs. 87.4%, respectively and stage IIB: 63.3 vs. 78.8%, respectively; P<0.05).

### Survival and lymph node involvement

**Figure 1.**
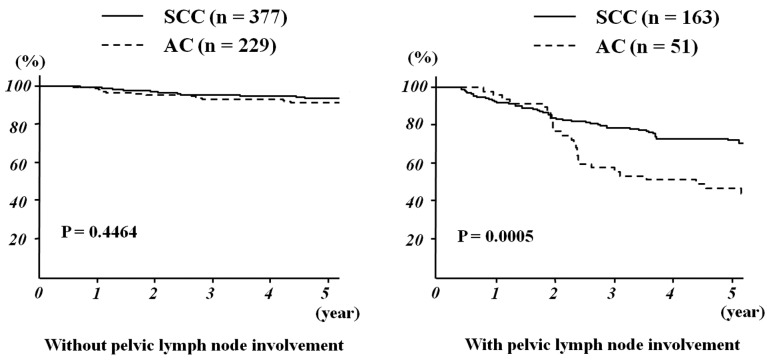
Five-year overall survival rate and pelvic lymph node involvement. Among patients with lymph node involvement, those with adenocarcinoma (AC) exhibited a significantly worse outcome compared to those with squamous cell carcinoma (SCC) (46.4 vs. 72.3%, respectively; P=0.0005), although the histological type did not affect the outcome of patients without lymph node involvement (AC, 91.2% and SCC, 93.9%; P=0.4464).

Patients with SCC exhibited significantly higher lymph node involvement compared to those with AC in stage IB1 (Table II). By contrast, lymph node involvement did not differ between AC and SCC in stages IB2 and II. Among patients with lymph node involvement, those with AC exhibited a significantly worse outcome compared to those with SCC (5-year OS: 46.4% vs. 72.3%, respectively; P=0.0005). However, the histological type did not significantly affect the outcome of patients without lymph node involvement (5-year OS: AC, 91.2% and SCC, 93.9%; P= 0.4464) (Fig. 1).

**Figure 2.**
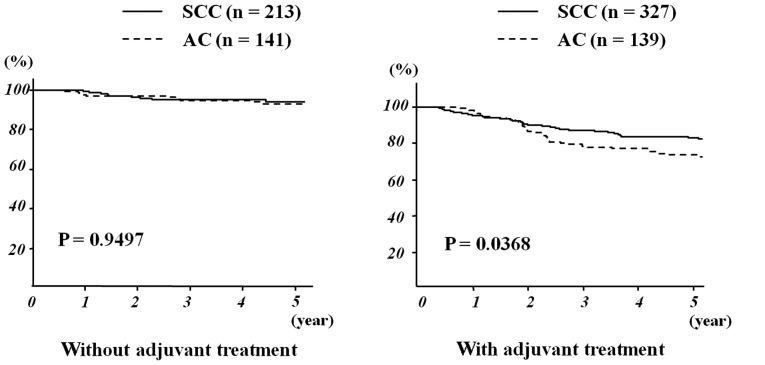
Five-year overall survival rate and adjuvant treatment. Among patients not receiving adjuvant treatment, those with adenocarcinoma (AC) exhibited a similar outcome to those with squamous cell carcinoma (SCC) (93.1 vs. 94.0%, respectively; P=0.9497). By contrast, among patients who underwent adjuvant treatment following radical hysterectomy, those with AC had a significantly worse outcome compared to those with SCC (73.7 vs. 83.1%, respectively; P=0.0368).

Among patients not receiving adjuvant treatment following radical hysterectomy, patients with AC exhibited an outcome similar to those with SCC (5-year OS: 93.1 vs. 94.0%, respectively; P= 0.9497) (Fig. 2). However, among patients who underwent adjuvant treatment, those with AC had a significantly worse outcome compared to those with SCC (5-year OS: 73.7 vs. 83.1%, respectively; P= 0.0368). Among stage II patients undergoing adjuvant treatment, patients with AC exhibited a significantly worse outcome compared to those with SCC (5-year OS: stage IIA, 50.0 vs. 86.9%, respectively, P= 0.0032; stage IIB, 61.1 vs. 75.5%, respectively, P= 0.037). However, the histological type did not significantly affect the outcome of patients with stage I disease (5-year OS: stage IB1: AC, 84.1 and SCC, 91.2%, P= 0.3374; stage IB2: AC, 74.6 and SCC, 76.1%, P=0.9127).

### Recurrence in patients undergoing adjuvant radiotherapy

In patients undergoing adjuvant treatment, there was no significant difference in the outcome between different treatments (5-year OS: CT, 79.4%; RT, 70.4%; RT + CT, 68.8%; P= 0.4522). In patients receiving adjuvant RT including CCRT, the 5-year OS was 70.4% in patients with AC and 81.7% in those with SCC (P=0.0858). Among patients receiving adjuvant RT, those with AC recurred more frequently compared to those with SCC (Table III). Furthermore, patients with AC recurred more frequently in the pelvic cavity, including the vaginal stump and/or pelvis, compared to those with SCC. By contrast, the histological type did not affect the incidence of recurrence in the paraaortic lymph nodes and/or distant recurrence.

## Discussion

Radiosensitivity is one of the important prognostic factors in the treatment of uterine cervical cancer; however, the number of clinical studies that have focused on patients with AC is limited. Eifel *et al* (16) reported that among 1,767 patients with stage I cervical cancer undergoing initial RT, those with AC had a worse prognosis compared to those with SCC, due to the higher incidence of distant metastasis in AC patients, although there were no significant differences in the rate of recurrence in the pelvic cavity between AC and SCC patients. By contrast, there was no significant difference in the incidence of distant recurrence between AC and SCC patients. According to a previous study, RT was less effective compared to surgery in patients with AC (13). Recently, Niibe *et al* (14) reported that the 5-year OS for stage IIIB patients with AC treated with high-dose intracavitary brachytherapy combined with external beam radiotherapy was 20.2%. The 5-year OS for stage IIIB patients with SCC has been reported to be 47.2–55.2% in Japan (17–19). These findings suggested that the radiosensitivity of AC was lower compared to that of SCC. In our series, among the patients undergoing adjuvant RT including CCRT, those with AC recurred significantly more frequently, particularly in the pelvic cavity, compared to those with SCC. Our data also indicated lower sensitivity to adjuvant RT in patients with AC. Consequently, RT including CCRT may not be the optimal adjuvant treatment for high-risk patients with AC following radical hysterectomy.

Pelvic lymph node involvement is an independent prognostic factor in patients with cervical cancer; however, it has not been elucidated whether patients with AC had a higher incidence of pelvic lymph node involvement compared to those with SCC. According to a previous study, the incidence of lymph node involvement in patients with SCC was significantly higher compared to those with AC and ASCC in stage IB (12.6 vs. 9.5%, respectively; P=0.0466) (8). By contrast, other studies suggested that there were no significant differences in the incidence of lymph node involvement between patients with AC and SCC (20,21). To the best of our knowledge, this is the first study to elucidate the incidence of pelvic lymph node involvement in stage IB1, IB2, IIA and IIB uterine cervical cancer patients. Furthermore, there was no significant difference in the incidence of lymph node involvement between patients with AC and SCC in stage IB2, IIA and IIB, although patients with SCC exhibited a significantly higher lymph node involvement compared to those with AC in stage IB1.

Among patients with pelvic lymph node involvement, those with AC had a significantly worse outcome compared to those with SCC. These results suggested that the poorer outcome of AC patients may be due to ineffective adjuvant treatment administered to patients with AC compared to those with SCC, rather than a higher incidence of lymph node involvement in AC compared to SCC. Furthermore, in the present study, the outcome of patients with stage I did not differ between AC and SCC; however, patients with AC exhibited a significantly worse outcome compared to those with SCC in stage II. Moreover, among patients undergoing adjuvant treatment following radical hysterectomy, the outcome of patients with AC was significantly worse compared to those with SCC. Consequently, patients with AC may have a poorer outcome compared to those with SCC when carcinoma invades beyond the uterine cervix, including stage II disease and pelvic lymph node involvement.

Certain studies suggested the significance of adjuvant CT for high- or intermediate-risk patients with cervical cancer. Takeshima *et al* (22) reported that adjuvant combination CT, containing bleomycin, vincristine, mitomycin and cisplatin, achieved a 5-year disease-free survival rate of 93.3% in 30 intermediate-risk patients and of 85.7% in 35 high-risk patients. Hosaka *et al* (23) reported that out of the 27 patients without multiple lymph node involvement who underwent adjuvant CT, only one patient recurred. In our series, adjuvant CT achieved the same outcome as adjuvant RT or concomitant RT and CT in patients with AC. These findings suggested the potential role of adjuvant CT for cervical cancer patients, particularly those with AC.

In conclusion, RT may not suffice as an adjuvant treatment for pathological risk patients with cervical AC following radical hysterectomy.

## Figures and Tables

**Table I.** Patient characteristics.

Variable	Histological type	
	AC (n=280)	SCC (n=540)
Age, years [mean (range)]	46.2 (18–84)	49.0 (19–84)
FIGO stage		
IB1	184	258
IB2	39	67
IIA	11	83
IIB	46	132
Adjuvant treatment		
Yes	139	327
RT or CCRT	69	258
CT	54	36
RT + CT	16	33
No	141	213

AC, adenocarcinoma; SCC, squamous cell carcinoma; FIGO, International Federation of Gynecology and Obstetrics; RT, radiotherapy; CCRT, concurrent chemoradiotherapy; CT, chemotherapy.

**Table II.** Incidence of pelvic lymph node involvement.

FIGO stage	Pelvic lymph node involvement		P-value
	AC (%)	SCC (%)	
IB1	9.8 (18/184)	16.7 (43/258)	0.0391
IB2	23.9 (11/39)	46.3 (31/67)	0.0667
IIA	36.4 (4/11)	34.9 (29/83)	0.9259
IIB	39.1 (18/46)	45.4 (60/132)	0.4566

FIGO, International Federation of Gynecology and Obstetrics; AC, adenocarcinoma; SCC, squamous cell carcinoma.

**Table III.** Recurrence in patients receiving adjuvant radiotherapy.

Pathological risk factor	AC (%)	SCC (%)	P-value
Recurrence outside RT field	15.9 (11/69)	14.3 (37/258)	0.7385
Recurrence within RT field	24.6 (17/69)	10.5 (27/258)	0.0022
Total recurrence	40.6 (28/69)	24.8 (64/258)	0.0096

Recurrence within RT field, recurrence in pelvic cavity including vaginal stump; recurrence outside RT field, recurrence in para-aortic lymph nodes and/or distant metastasis. AC, adenocarcinoma; SCC, squamous cell carcinoma; RT, radiotherapy.
